# Word balloon catheter for Bartholin’s cyst and abscess as an office procedure: clinical time gained

**DOI:** 10.1186/s13104-015-1795-3

**Published:** 2016-01-06

**Authors:** Vincent Boama, Joanne Horton

**Affiliations:** Department of Obstetrics and Gynecology, Sidra Medical and Research Center, Po Box 26999, Doha, Qatar; Department of Obstetrics and Gynecology, Hampshire Hospitals NHS Trust, Basingstoke, Hampshire UK

**Keywords:** Word balloon catheter, Marsupialization, Bartholin’s cyst, Bartholin’s abscess, Office procedure

## Abstract

**Background:**

Around 2 % of women develop a Bartholin’s cyst or abscess at least once in their life time. The use of Word balloon catheter as an office procedure for the management of Bartholin’s cyst and abscess has been well described and indicates high patient acceptance, low short-term recurrence rates and reduced cost. In most of the reported studies, the reduced costs are attributed to savings from equipment cost, operating theatre costs and health personnel costs. An evaluation of the actual clinical time gained with this office procedure has not been reported and hence the rationale for this study. This study was conducted from December 2011 to January 2014 on 35 patients. An initial retrospective clinical audit of 14 cases of marsupialization under general anesthetic between December 2011 and December 2012 was performed. The findings were compared with a subsequent prospective observational service evaluation of 21 consecutive patients between December 2012 and January 2014.

**Results:**

Compared to marsupialization under general anesthetic, the mean clinical time gained from admission to insertion of Word balloon catheter as an office procedure is 15 h and 40 min and the mean clinical time gained from admission to discharge is at least 24 h. There were very few minor complications and no major complications in the Word catheter group compared to the marsupialization group.

**Conclusions:**

There is a clinically significant time gained with the use of Word balloon catheter as an office procedure compared to marsupialization under general anesthetic for Bartholin’s cyst and abscess. The findings from our study could assist other units that want to adopt this procedure justify the efficiency savings in terms of clinical time gained when a business case is submitted. Further studies are needed to investigate and address the underlying causes for the delays encountered when marsupialization under general anesthetic is chosen by patients.

**Electronic supplementary material:**

The online version of this article (doi:10.1186/s13104-015-1795-3) contains supplementary material, which is available to authorized users.

## Background

Around 2 % of women develop a Bartholin’s cyst or abscess at least once in their lifetime [1, 2]. There are many treatment modalities that have been described and these include marsupialization [3, 4], silver nitrate application [5], carbon dioxide laser vaporization [6], use of Word catheter [7] and aspiration and alcohol sclerotherapy [8], amongst others. The authors of a systematic review concluded that none of these treatment modalities appear to be superior over another with respect to success and recurrence rates [9]. A retrospective study published in 2013 which investigated the clinical course and causative microorganisms of Bartholin gland abscesses found a 37 % recurrence rate and also showed a 43.7 %* E. coli* culture positivity [10]. The use of Word catheter as an office procedure is currently gaining much popularity due to cost savings and other patient benefits [11–13]. Clinical time gained from the use of Word balloon catheter as an office procedure compared to the standard practice of marsupialization under general anesthetic has not been evaluated. With the ever-increasing need for efficiency savings in health care globally, the need to determine best ways of achieving these is paramount.

## Objectives

This service evaluation was performed to determine if there is a clinically significant time gain from the use of Word balloon catheter as an office procedure compared to the standard practice of marsupialization under general anesthetic. In our study, we focus on the time aspect of Word balloon catheter use in patients with Bartholin’s cyst and abscess, since quality of life issues, implementation issues, recurrence rates and complication rates as well as costs have already been researched and published.

## Methods

This study was conducted from December 2011 to January 2014 in a United Kingdom District General Hospital. The use of Word balloon catheter as an office procedure became an available treatment option for patients with a Bartholin’s cyst or abscess from December 2012 in the study hospital. Prior to that all such patients were offered marsupialization under general anesthesia as the only surgical treatment. An initial retrospective clinical audit of 14 cases of marsupialization under general anesthetic between December 2011 and December 2012 was performed. The findings were compared with a subsequent prospective observational service evaluation of 21 consecutive patients between December 2012 and January 2014. Following thorough clinical assessment and counselling regarding treatment options, these 21 patients were offered marsupialization under general anesthetic in the operating theatre or insertion of Word catheter under local anesthetic as an office procedure. From December 2012 to December 2013, 9 patients opted for marsupialization under general anesthetic and 12 patients opted for Word balloon catheter insertion under local anesthetic. Patients undergoing Word catheter balloon insertion were given full explanation of the procedure and patient information leaflets before obtaining written consent. The procedure technique and equipment list as well as the patient information leaflet can be found as Additional files 1 and 2 respectively to this manuscript. The Questionnaire administered post insertion of Word Balloon Catheter is attached as Additional file 3. The retrospective clinical audit and the prospective service evaluation were both registered with the Institutional Audit and Clinical Effectiveness Office. This study was exempt from the ethical approval process by the Institutional Review Board because it was a clinical audit and a service evaluation.

## Results

See Table 1 for results. The total number of patients recruited was 35. In the retrospective audit group of patients who underwent marsupialization mean age was 32 years (range 21–47 years). 4 (29 %) had Bartholin’s cysts and 10 (71 %) had Bartholin’s abscess. Mean admission to surgery time was 9.98 h (range 0.5–33 h) and mean admission to discharge time was 24.5 h (range 7–53 h). In 28.5 % of these cases, there were significant delays from time of decision to patients arriving in theatre. Ninety-five percent of these delays were due to a busy emergency operating theatre list. The remaining five percent were due to unavailability of a physician to perform the procedure due to more urgent clinical work at that time.

**Table 1:** Retrospective audit and prospective service evaluation results

	Retrospective audit group (n = 14)	Prospective service evaluation group (n = 21)	
	Marsupialization (n = 14)	Marsupialization (n = 9)	Word Catheter (n = 12)
Mean age (range)	32 years (21–47 years)	39 years (21–57 years)	34 years (22–51 years)
Cyst	4 (29 %)	4 (19 %)	
Abscess	10 (71 %)	17 (81 %)	
Mean admission to surgery interval (range)	9.98 h (0.5–33 h)	16 h (2.5–24 h)	20 min (10–40 min)
Mean admission to discharge interval (range)	24.5 h (7–53 h)	31 h (9.5–48 h)	40 min (20–90 min)

In the prospective study group, mean age was 39 years (range 21–57), 4 (19 %) were Bartholin’s cysts and 17 (81 %) were Bartholin’s abscesses. In the patients who had marsupialization in the prospective study group mean admission to surgery was 16 h (range 2.5–24 h), mean admission to discharge was 31 h (range 9.5–48 h). In the patients who had Word balloon catheter mean admission to surgery was 20 min (range 10–40 min), mean admission to discharge was 40 min (range 20–90 min).

In the Word balloon catheter group, 7 (57 %) found the procedure highly acceptable, 7 (58 %) would have the procedure again if there were a recurrence and 12 (100 %) would recommend the procedure to family and friends.

In the Word catheter group, there were no treatment failures described as expulsion of Word balloon catheter within 24 h. We had no cases of readmissions. There were no cases of post insertion abscess or cellulitis. Only one patient reported pain and discomfort at home over the 4 week period. In three patients, the Word catheter was expelled but in all three, the expulsion occurred more than 3 days post insertion. They contacted the team by telephone for advice without needing to attend the hospital. There were no cases of recurrence. The reasons are likely to be due to the small number of cases in our study and also due to the short follow up period of 4–6 weeks.

In the marsupialization under general anesthetic group, there was one case of severe laryngospasm at intubation requiring intervention from a senior Consultant Anesthetist. There was one case of intraoperative pyrexia attributed to anesthetic drugs. There was one case of unexplained post operative hypotension and oxygen desaturation while on the recovery ward requiring fluid and Oxygen resuscitation. Patient satisfaction data was not sought from the marsupialization group, as the initial arm was a retrospective clinical audit and marsupialization was standard clinical practice.

## Discussion

The findings from this study support existing publications [11–13], which show high patient acceptance and cost benefits of Word catheter as an office procedure compared to marsupialization under general anesthetic. Our study also confirms a low rate and minor nature of complications and high patient acceptance with the use of Word balloon catheter as an office procedure in patients with Bartholin’s abscess and cysts. Compared to the high recurrence rate of 37 % found in another study [10], our study did not find any recurrence of Bartholin’s cysts or abscesses, most likely because of the small number of cases and the short follow up period of 4–6 weeks. The key additional information contributed by this study is the actual clinical time gained by the adoption of the office procedure as compared to marsupialization under general anesthetic. We can theoretically deduce there are efficiency savings attributable to the clinical time gained, avoidance of operating theatre and equipment and reduced need for healthcare personnel when Word catheter balloon is used in an office setting compared to marsupialization under general anesthetic. This could justify the adoption of this office procedure by other units.

## Conclusions

There is a clinically significant time gained with the use of Word balloon catheter as an office procedure compared to marsupialization under general anesthetic for Bartholin’s cyst and abscess. The findings from our study could assist other units who want to adopt this procedure justify the efficiency savings in terms of clinical time gained when a business case is submitted. Further studies are needed to investigate and address the underlying causes for the delays encountered when marsupialization under general anesthetic is chosen by patients.

## Additional files

10.1186/s13104-015-1795-3 Word balloon catheter insertion procedure technique and equipment list.
10.1186/s13104-015-1795-3 Patient information leaflet “Word Balloon Catheter Insertion for Bartholin’s Cyst or abscess”.
10.1186/s13104-015-1795-3 Word balloon catheter patient questionnaire.
