# Supplementary material for: Word balloon catheter for Bartholin’s cyst and abscess as an office procedure: clinical time gained
Source: BMC Res Notes. 2016 Jan 6;9:13. doi: 10.1186/s13104-015-1795-3 (PMC4702305; doi:10.1186/s13104-015-1795-3)
Supplement: Supplementary file 1 — 10.1186/s13104-015-1795-3 Word balloon catheter insertion procedure technique and equipment list. [file 13104_2015_1795_MOESM1_ESM.doc]

**Use of COOK Word Catheter for Bartholin’s Duct Cyst and Gland Abscess**

**Introduction:**

2% of women develop a Bartholin’s duct cyst/ gland abscess in their life. Traditionally, if symptomatic, they are managed with marsupialisation under general anaesthetic. An alternative to this is a simple treatment that can be performed in EGU under local anaesthetic.

**Principle:**

The catheter is a balloon tipped catheter that is positioned into the cyst/abscess cavity and acts as a foreign body in the wound preventing closure, thus resulting in the formation of an epithelialised fistula or sinus track. When the catheter is removed, the newly created duct will shrink and the gland will return to its normal function.

**Contra-indications to use of Word Bartholin’s catheter**:

Patient declines.

**Procedure:**

1. Informed written consent after patient has read the Patient Information leaflet ( Appendix B-1)
2. Benefits-to treat abscess/cyst
3. Complications-infection, bleeding, discomfort, pain. Catheter may fall out. Abscess/cyst may recur.
4. Prepare sterile field with the woman in lithotomy position and ensure good lighting.
5. Administer local anaesthetic ( eg 2ml 1% Prilocaine Hydrochloride-Citanest-in dental syringe) and allow 2 minutes for this to take effect.
6. Grasp the wall of the cyst/abscess with a small forceps and using a No.11 blade, make a 5mm incision ( stab) into the cyst or abscess. The incision should be within the introitus external to the hymenal ring in the area of the duct orifice. It is important to grasp the cyst wall before the incision is made otherwise the cyst can collapse and a false tract may be created. If the incision is too large, the Word catheter may fall out.
7. Insert a charcoal swab to ensure any loculation broken down and send for MC&S. Express as much fluid/pus from the cavity as possible.
8. Insert the Word catheter and inflate the balloon tip with 2-3ml saline solution injected through the hub of the catheter. The inflated balloon allows the catheter to remain within the cavity of the cyst or abscess.
9. The free end of the catheter can be placed into the vagina
10. Unless there is evidence of cellulitis, antibiotics ( see hospital antibiotic policy) whilst awaiting cultures.
11. Leave in place for 4 weeks to allow epithelialisation.
12. Discuss and give Patient Information leaflet following insertion of Bartholin’s ( Appendix B-2)
13. Complete discharge summary. Include attention for GP for a 6 weeks review.
14. Prescribe simple analgesia ( paracetamol & NSAIDS) and antibiotics if required.
15. Ensure Patient has a date and time booked to return in 4 weeks for removal of Word catheter at the emergency gynaecology Unit.

**Follow up:**

1. When patient returns in 4 weeks time for removal of Word catheter on the emergency gynaecology unit, deflate the Word catheter by removing the 2-3ml fluid after explaining the procedure and obtain verbal consent. No anaesthetic is required for this.
2. Answer any questions that the patient may have and Provide the Patient Audit Questionnaire ( Appendix C) for completion.
3. Patient can then be discharged home . Remind Patient to contact GP for a review in 2 weeks time ( 6 weeks from insertion of Word catheter).

**Equipment List:**

Sterile gloves

Cleaning solution ( eg Chlorhexidine)

Dental syringe and needle

Local anaesthetic ( eg 2ml 1% Prilocaine HCL)

Word catheter

3ml saline solution

25 gauge, 1 inch needle with 3ml syringe for inflating balloon

NO. 11 scalpel

Small forceps

Gauze pads

Plain swab

**Still need help:**

Contact Dr Vincent Boama if you need training on how to perform the procedure or if you have any queries.
