# Supplementary material for: Word balloon catheter for Bartholin’s cyst and abscess as an office procedure: clinical time gained
Source: BMC Res Notes. 2016 Jan 6;9:13. doi: 10.1186/s13104-015-1795-3 (PMC4702305; doi:10.1186/s13104-015-1795-3)
Supplement: Supplementary file 2 — 10.1186/s13104-015-1795-3 Patient information leaflet “Word Balloon Catheter Insertion for Bartholin’s Cyst or abscess”. [file 13104_2015_1795_MOESM2_ESM.docx]

## Patient information sheet

## Word balloon Catheter for the treatment of Bartholin’s cyst or abscess.

**This leaflet is for women who have been offered a Word catheter ( a very small inflatable balloon) to treat a Bartholin’s cyst or abscess. This advice is based on guidance from NICE ( National Institute for Health and Clinical Excellence).**

**This leaflet is written to help you decide whether to agree ( consent) to it or not. It does not describe the procedure in detail. A member of the healthcare team offering you this treatment option would give you full information and advice about these.**

There is a Bartholin’s gland at each side of the entrance to the vagina. During sexual arousal they produce lubrication that enters the vagina through a small duct ( tube) from each gland. If the duct becomes blocked the gland can fill with mucus and a cyst ( fluid filled lump) can occur forming a Barthlin’s cyst. A Bartholin’s abscess can occur if the gland or cyst becomes infected. The symptoms may include tenderness and pain, fever, lump at the entrance to the vagina and pain or discomfort during sex.

Treatment for Bartholin’s cyst or abscess is usually by warm baths, compresses and pain killers to relieve the symptoms. Antibiotics may be given if there is an infection. If the cyst or abscess is causing symptoms and does not respond to these treatments, surgical treatment may be used to drain it.

The two surgical treatments that we offer at Hampshire Hospitals NHS Trust are : 1**) Marsupialisation of Bartholin’s cyst or abscess under general anaesthetic** ( putting you to sleep) in theatre . This is making a permanent opening that allows the gland to drain whilst you are under general anaesthetic .

2**) Word balloon catheter insertion for Bartholin’s cyst or abscess using local anaesthetic** .This is making a very small cut into the cyst or abscess using local anaesthetic and inserting a small inflatable balloon to help the gland drain freely. The local anaesthetic is very similar to the ones given by a dentist for tooth extraction.

**Is it safe to use balloon catheter insertion for Bartholin’s cyst or abscess?** Yes. NICE has said that this procedure is safe enough and works well enough for use in the NHS and can be offered routinely as treatment option for a Bartholin’s cyst or abscess provided that doctors are sure that: i) the patient understands what is involved and agrees to treatment and ii) the results of the procedure are monitored.

**How does the balloon catheter work?** There is a small balloon that is blown up with a small amount of fluid that sits inside the cyst or abscess wall. This stops the catheter falling out. The catheter allows the cyst or abscess fluid to drain.

**How long does the catheter stay in for?** The catheter stays in for 4 weeks. You will be given an appointment to return to the hospital to have the catheter removed.

**Why does the catheter have to stay in for so long?** It has to stay in long enough for the skin to heal around the catheter. When the catheter is removed a tiny opening will remain and allow the gland to drain and reduce the risk of future recurrence.

**How is the catheter removed?** We simply remove the fluid from the balloon using a syringe and gently pull the catheter out. This may cause very mild discomfort only. The removal of the catheter will take place in one of the clinic rooms. It is quick and does not require any anaesthetic.

**What are the benefits I might get?** The benefits include the avoidance of hospital admission, avoidance of a general anaesthetic and immediate return to your home to continue with your normal activities.

**What are the risks of the procedure?** The risks of this procedure are minor and most are uncommon. They include pain or discomfort at the time of the procedure, a small risk of bleeding or infection and a very rare risk of an abscess formation.

**Can the cyst or abscess recur after this procedure?** Yes . The chances of the cyst or abscess growing back after this procedure is 3-17 cases for every 100 cases.

**Am I going to be sore when the local anaesthetic wears off?** Yes, it is likely to be a little bit uncomfortable where we made the cut for a few days. You can take paracetamol or Ibuprofen ( if you are not allergic to them). You can speak to your GP if you need stronger pain killers.

**Can I have a bath or shower with the catheter in?** Yes, you can continue to was as normal.

**Can I have sex with the catheter in ?** Yes, as soon as you feel comfortable enough.

**Can I use tampons for my periods?** Yes, you can continue as normal. Just be careful not to pull on the catheter when removing the tampon.

**What happens if the catheter falls out?** Sometimes the catheter can fall out. If this happens, do not worry, give us a call on the numbers below and we will arrange to see you again in the hospital to discuss and plan further treatment if necessary.

**What happens if something goes wrong or if I have more questions when I am at home?** Do give us a ring on the numbers below to speak to a member of the gynaecology team for advice.

**Are there alternative procedures?** Yes. I member of the team will discuss and explain the alternative procedure of Marsupialisation under general anaesthetic to you.

**What may happen if I don’t have the procedure?** You have the right to refuse any form of treatment that is offered to you. A member of the gynaecology team will discuss and explain further options of management if you decline to have the Word catheter procedure.

**Why do I have to complete the Patient Questionnaire after the Word catheter has been removed**? The completion of the Patient questionnaire is voluntary and anonymous ( cannot be traced back to you). It is an opportunity for feed- back from people who have had this procedure. This information would be used as part of the clinical audit on the procedure and may help us improve our service to all patients in future.

## Contact telephone numbers are:

### Emergency Gynaecology Unit at Basingstoke Hospital: 01256312766 ( Mon-Fri 08:30- 20:00)

## Gynaecology Ward ( Basingstoke Hospital): 01256313583/4 ( Mon –Sun. 24 hours)
