# Supplementary material for: Word balloon catheter for Bartholin’s cyst and abscess as an office procedure: clinical time gained
Source: BMC Res Notes. 2016 Jan 6;9:13. doi: 10.1186/s13104-015-1795-3 (PMC4702305; doi:10.1186/s13104-015-1795-3)
Supplement: Supplementary file 3 — 10.1186/s13104-015-1795-3 Word balloon catheter patient questionnaire. [file 13104_2015_1795_MOESM3_ESM.doc]

**Patient Questionnaire following insertion of Word catheter for Bartholin’s cyst/abscess**

This questionnaire must be given to every patient who has had a Word catheter to treat a Bartholin’s cyst or abscess. This questionnaire must be completed by the patient after removal of the Word catheter.

1. **Overall, how did you find the procedure?**
2. Highly acceptable
3. b) Just acceptable
4. c) Not acceptable
5. d) Not sure
6. **Did you find the procedure painful ? ( score of 0-10 where 0 is no pain and 10 is the worst pain)**
7. During insertion of the Word catheter: ……………….
8. During the 4 weeks at home:………………..
9. During removal at the end of 4 weeks:……………………
10. **Did you encounter any problems or difficulties during the procedure?**
11. No
12. Yes…………………………………………………………………………………………………………………………………………………………………………………………………………………………………………………………………
13. **Did you encounter any problems or difficulties with the catheter at home?**
14. No
15. Yes:…………………………………………………………………………………………………………………………………………………………………………………………………………………………………………………………………
16. **Did the catheter fall out at home?**
17. No
18. Yes:……………………………………….
19. **Did you encounter any problems or difficulties during sex because of the catheter**?
20. No
21. Did not have sex
22. Yes:……………………………………………………………………………………………………………………………………………………………………
23. **Would you have Word catheter for treatment of Bartholin’s cyst or abscess again in future:**
24. Yes
25. No:………………………………
26. Not sure
27. **Would you recommend this procedure to a friend or family member ?**
28. Highly recommend
29. Just recommend
30. Not recommend……………………………………………………….
31. Not sure

**Any comments or feedback about the procedure** ? :…………………………………………………………………………………………………………………………………………………………………………………………………………………………………………………………………………………………………………………………………………………………………………………………………………………………………………………………………………………………………………………………………………………………………………………………………………………………………………………………………………………………………………………………………………………………………………………………….

**Thank you**
